# Mechanism of Action of Plant Extracts in Preventing Post-Weaning Diarrhea in Piglets: A Review

**DOI:** 10.3390/vetsci13040312

**Published:** 2026-03-25

**Authors:** Xiaohan Jiang, Haibo Xiao, Peng Huang

**Affiliations:** 1 Hunan Key Laboratory of Traditional Chinese Veterinary Medicine, College of Veterinary Medicine, Hunan Agricultural University, Changsha 410127, China; jiangxiaohan@stu.hunau.edu.cn; 2 College of Animal Science and Technology, Hunan Agricultural University, Changsha 410127, China; 1241406911@stu.hunau.edu.cn

**Keywords:** post-weaning diarrhea, plant extracts, gut microbiota, intestinal barrier, oxidative stress

## Abstract

Post-weaning diarrhea (PWD) in piglets is a common multifactorial disease in global swine production. Traditional prevention and control strategies relying on high-dose zinc oxide and antibiotics face limitations due to antimicrobial resistance and environmental concerns. This review systematically elucidates the mechanisms of action of plant extracts as alternative interventions against PWD, focusing primarily on categories such as polysaccharides, polyphenols, essential oils, and alkaloids. This review discusses how plant extracts exert their effects through various mechanisms, including improving feed intake, enhancing antioxidant and immune capacities, modulating gut microbiota, repairing intestinal barrier function, and inhibiting pathogens—to alleviate diarrhea induced by weaning stress and pathogenic infections. The findings indicate that plant extracts offer advantages such as multi-target effects, natural origin, and a low propensity for inducing resistance, highlighting their promising potential as alternatives to conventional additives. However, challenges related to compositional complexity, stability, and bioavailability require further investigation and optimization.

## Simple Summary

This article provides a comprehensive review the major pathophysiological mechanisms underlying post-weaning diarrhea (PWD) in piglets under weaning stress and the intervention effects of plant-derived bioactive compounds. Weaning stress markedly increases piglet susceptibility to pathogens such as enterotoxigenic *Escherichia coli* (ETEC) by inducing oxidative stress, intestinal barrier damage, immune dysregulation, and microbiota dysbiosis. Evidence indicates that plant extracts, including, polysaccharides, polyphenols, essential oils, and alkaloids, can effectively alleviate the onset and progression of PWD through multi-target and synergistic mechanisms, such as increasing feed intake, enhancing antioxidant defense, modulating immune and inflammatory responses, repairing the intestinal barrier, and reshaping the gut microbiota. Overall, plant-derived bioactive substances exhibit significant advantages in strengthening the endogenous intestinal defense capacity of weaned piglets and demonstrate great potential as alternatives to in-feed antibiotics and zinc oxide for achieving sustainable and healthy pig production.

## 1. Introduction

PWD in piglets is a common multifactorial disease in global swine production, primarily occurring within 14 days post-weaning [1]. Clinically, affected piglets present with diarrhea, often characterized by the excretion of yellow or gray liquid during the first week, which typically lasts for three to five days, and may persist up to two weeks [2]. Diarrhea leads to severe dehydration, weight loss, and growth retardation. In severe cases, PWD can cause sudden death [3], with mortality rates reaching as high as 25% in affected pens [2], posing a serious threat to piglet health and welfare. PWD not only significantly reduces piglet survival and growth performance, but also prolongs the finishing period, increases the feed conversion ratio, and raises the costs of veterinary intervention and management, thereby exerting a profound negative impact on the economic efficiency of pig production. Particularly under large-scale and intensive production systems, outbreaks of PWD often present with group occurrence and recurrence, further increasing production pressure on the farming system.

The etiology of PWD is complex, resulting from the combined effects of weaning stress, nutritional changes, gut dysfunction, and pathogenic infections. Early post-weaning, due to underdeveloped intestinal function, the balance between intestinal structure, digestive function, and the apoptosis and proliferation of epithelial cells is impaired following the sudden weaning [4]. Abrupt dietary transition at weaning (from milk to solid feed) can disrupt intestinal barrier function and mucosal immunity and reshape the gut microbiota in piglets, thereby predisposing them to PWD; notably, ETEC can more readily colonize the intestine after early weaning [5]. Furthermore, weaning interrupts lactogenic passive protection; once separated from the sow, piglets no longer receive maternal antibodies present in the sow’s milk, which contributes to their increased vulnerability to post-weaning diarrhoea [6]. During this critical physiological transition, structural and functional alterations such as villus atrophy, crypt deepening, and increased intestinal permeability often occur simultaneously, resulting in a marked decline in intestinal barrier function and creating favourable conditions for the adhesion, colonisation, and proliferation of pathogenic microorganisms. Damage to the intestinal barrier not only impairs nutrient absorption but also induces local and systemic inflammatory responses, thereby further amplifying the negative effects induced by weaning stress.

Additionally, the weaning process requires piglets to undergo significant behavioral adaptations, including environmental changes and social structure reorganization. These changes often trigger stress-related behaviors, such as reduced feeding and drinking time, leading to decreased voluntary feed and water intake [3], and potentially harmful behaviors, such as tail biting, which have the highest incidence in weaned piglets [7]. Psychological and social stress affects intestinal function through the neuro–endocrine–immune axis, promoting increased secretion of stress hormones (e.g., cortisol), thereby suppressing immune responses, disrupting intestinal homeostasis, and aggravating intestinal inflammation and the risk of diarrhoea. This indicates that the occurrence of PWD is not only a digestive system disease, but also a comprehensive consequence of multisystem imbalance.

In the past, high doses of zinc oxide (ZnO) have been widely used in pig farming to prevent and reduce the occurrence of PWD. However, starting in June 2022, the European Union banned the use of ZnO, and major pig-raising countries and regions, including China, have set clear regulations on the use of ZnO in pig feed, particularly imposing strict limits on high-dose ZnO use [8]. Antibiotics are effective in treating *E. coli*-induced diarrhea in piglets, but their efficacy is severely limited by the emergence and spread of antimicrobial resistance [9]. Moreover, antibiotic-resistant bacteria in food animals can be transmitted to humans along the food production chain [10], posing a significant threat to human health. Consequently, national and international strategies and action plans have been implemented to address antimicrobial resistance [11,12]. Against this background, identifying safe, effective, and sustainable alternatives to antibiotics and high-dose ZnO has become one of the key research priorities in animal nutrition and veterinary medicine. Alternative strategies not only need to demonstrate reliable efficacy in controlling diarrhoea, but also should take into account multiple goals, including animal health, food safety, and environmental protection.

In recent years, various plant extracts have been studied for improving weaning transition and alleviating intestinal diseases [13]. Numerous studies suggest that various plant extracts, such as essential oils, polyphenols, and polysaccharides [14,15,16], can effectively mitigate the symptoms of PWD. These plant extracts are widely sourced and diverse in structure and function, and typically exert their effects through multiple mechanisms, including antioxidation, anti-inflammation, antibacterial activity, modulation of the gut microbiota, and enhancement of intestinal barrier function, demonstrating an integrated regulatory advantage of “multi-target and multi-pathway” actions. Compared with conventional drugs, plant extracts are less likely to induce antimicrobial resistance during long-term application and are more consistent with the concept of green production and sustainable development.

To better understand potential intervention strategies, it is first necessary to clarify the major pathophysiological mechanisms underlying PWD in piglets. This review aims to summarize several common types of plant extracts to better explain their regulatory effects on the causes of PWD. This review explores the effects of different plant extracts on alleviating weaning stress, improving intestinal structure and function, regulating immune responses, and reshaping the gut microbiota. It aims to provide a theoretical basis for the use of plant-derived feed additives in the prevention and control of PWD in piglets, while also offering insights for future research and practical applications in the industry.

## 2. Causes of PWD in Piglets

### 2.1. Impairment of Intestinal Barrier Structure and Function

Weaning is considered one of the most intense physiological and psychological stress events during early life. The sudden changes in nutrient sources, environmental conditions, and social structure exert profound effects on the intestine via the neuro-endocrine-immune axis, leading to a series of structural and functional disorders.

The transition from sow’s milk to dry, plant-based feed, which contains high levels of antinutritional factors and is more difficult to digest, coupled with an immature digestive enzyme system, often reduces voluntary feed intake and aggravates nutrient deficiency [13]. Early post-weaning feeding is a key driver of intestinal development, and insufficient intake has been associated with intestinal inflammation and characteristic lesions such as villus atrophy and increased crypt depth [17]. These morphological changes directly reduce the effective absorptive area of the small intestine and disrupt the balance between epithelial cell turnover and differentiation, further weakening nutrient absorption and barrier defense. Therefore, promoting feed intake after weaning is crucial for preventing and alleviating the occurrence of PWD.

Besides the physical barrier formed by tight epithelial cell junctions, the intestine is further protected by a mucus layer produced by goblet cells, which are closely related to the immune system [18]. However, Tang et al. observed that weaning reduces the proliferation of goblet cells and mucus secretion in the intestine [4]. Notably, Boudry et al. observed that pigs with higher feed intake exhibited an increased number of goblet cells later, indirectly indicating potential early barrier damage after weaning and suggesting its repair potential [19]. Therefore, improving feed intake through nutritional interventions, thereby promoting goblet cell function recovery, is an important approach to alleviating weaning-induced intestinal barrier damage.

### 2.2. Pathogenic Microbial Colonization and Infection

Colonization and infection by pathogenic microorganisms are the direct triggers of PWD. Weaning stress causes a decline in intestinal barrier function and a weakened immune defense, which creates favorable conditions for pathogen adhesion, colonization, and proliferation in the intestine, thereby significantly increasing the risk of PWD.

Among numerous pathogens, Enterotoxigenic *Escherichia coli* (ETEC) has been clearly identified as the most important causative agent of PWD [20]. The pathogenicity of ETEC depends on its virulence factors, which mainly include fimbrial adhesins and enterotoxins. Among the different adhesins, F4 (K88) is the most frequently detected type, followed by F18. Most ETEC strains associated with PWD carry F4 (K88) or F18 fimbriae, which mediate adhesion to porcine intestinal epithelial cells, followed by enterotoxin production that disrupts fluid and electrolyte homeostasis and induces secretory diarrhea [21,22]. This process not only causes severe loss of water and nutrients but also further weakens the growth performance and immune status of piglets. In addition, studies from South Korea have further confirmed that F4 and F18 fimbriae are closely associated with PWD and edema disease (ED), and have revealed their specific antigenic variations [23]. Under field conditions, mixed infections and variation in pathogen load can occur, and disease expression ultimately reflects the interaction between pathogen virulence and host barrier–immune status.

### 2.3. Gut Microbiota Dysbiosis

During the weaning period, abrupt changes in environment, nutrition, and physiological status markedly affect gut microbial colonization and community structure, thereby disrupting microbial ecological balance. Such dysbiosis may precede PWD and reflect disease susceptibility. Dou et al. found that at postnatal day 7 (PND), which is approximately four weeks before the occurrence of weaning-associated diarrhea, significant differences in fecal microbiota were already evident between pigs that later developed diarrhea (D-type) and healthy pigs (H-type). At PND7, H-type pigs exhibited lower microbial evenness but higher abundances of *Prevotellaceae*, *Lachnospiraceae*, *Ruminococcaceae*, and *Lactobacillaceae* compared with D-type pigs [24]. These findings suggest that early colonization patterns are associated with a lower risk of subsequent diarrhea, although causality remains to be established.

These taxa are closely linked to fermentation of complex carbohydrates, production of short-chain fatty acids, and maintenance of intestinal immune homeostasis. *Prevotella* and *Roseburia* contribute to degradation of complex polysaccharides and generation of short-chain fatty acids that support host energy supply and intestinal health [25]. Consistently, Singh et al. demonstrated that the abundance of *Prevotella* and *Roseburia* is negatively correlated with *Escherichia coli*-induced intestinal infections [26]. In contrast, Proteobacteria is widely regarded as a biomarker of dysbiosis and intestinal inflammation, and other microbial groups or metabolites may aggravate barrier dysfunction by disrupting the mucus layer, damaging epithelial cells, or inducing inflammatory responses, including taxa with mucin-degrading or hydrogen-sulfide-producing potential [24].

Importantly, the relationship between dysbiosis and diarrhea is bidirectional. Diarrhea episodes can rapidly alter luminal nutrient availability, pH, oxygen tension, and intestinal transit time, thereby secondarily reshaping microbial communities and their metabolite profiles [5]. Consequently, microbiota shifts observed during or after PWD should be interpreted with caution, as they may represent causal mediators contributing to symptom improvement or merely downstream markers of gut recovery. To clarify this distinction, studies should incorporate causal strategies such as microbiota depletion followed by fecal microbiota transplantation [27,28], and, where relevant, metabolite-focused validation such as SCFA add-back or infusion [29]. Based on these considerations, the anti-diarrheal effects of plant extracts are discussed here as arising from non-mutually exclusive routes, including direct actions on pathogens and or toxins, direct modulation of the gut epithelium and immune responses including epithelial secretion, and indirect effects mediated by fermentation products or microbially transformed metabolites.

### 2.4. Oxidative Stress

Weaning is a strong physiological and psychological stressor. Stressors such as maternal separation, transportation, regrouping, fighting and establishment of new social hierarchies, and vaccination can lead to excessive production of reactive oxygen species (ROS) in piglets [30]. When the rate of ROS generation exceeds the scavenging capacity of the endogenous antioxidant system, oxidative stress occurs and redox homeostasis is disrupted [31]. Lipid peroxidation is a key hallmark of this process, and malondialdehyde (MDA), is commonly used as a biomarker to assess the degree of oxidative stress in vivo [32]. Oxidative stress can damage cellular membranes and other macromolecules, further impairing normal cellular function and intestinal integrity [31].

Oxidative stress further aggravates intestinal barrier damage and is tightly intertwined with intestinal inflammation, forming a vicious cycle. Excessive ROS can disrupt epithelial cell membrane structure, suppress tight junction protein expression, and impair cellular renewal and repair capacity. Studies have shown that certain pathogenic bacteria can directly or indirectly induce ROS production, thereby exacerbating intestinal inflammation [33]. Increased permeability then creates favorable conditions for pathogen invasion, while invading pathogens further stimulate pro-inflammatory cytokine release and ROS generation, resulting in a self-amplifying pathological cycle [33].

The host counters oxidative stress through a complex antioxidant defense network, in which the nuclear factor erythroid 2–related factor 2 (Nrf2) signaling pathway plays a central role. Under oxidative stress, Nrf2 is activated and translocates into the nucleus, initiating the transcription of multiple antioxidant enzymes, including glutathione peroxidase (GSH-Px), to maintain cellular redox homeostasis [32]. Nutritional interventions targeting this pathway have shown promising results. Studies have demonstrated that Lactobacillus rhamnosus LB1 and Parabacteroides distasonis HNAU0205 derived from Ningxiang pigs can alleviate oxidative damage induced by ETEC challenge by regulating signaling pathways such as Nrf2 and enhancing the activities of antioxidant enzymes including GSH-Px [32,34]. These findings suggest that alleviating weaning stress–associated oxidative damage through regulation of host antioxidant signaling pathways may represent an important entry point for the prevention and control of PWD.

### 2.5. Systemic Immune Dysregulationn

Weaning stress and pathogenic infection can lead to immune dysfunction in piglets, characterized by suppressed humoral immune responses together with excessive activation of pro-inflammatory reactions. The weaning period represents an immunological gap during which maternally derived passive immunity wanes while the piglet’s active immune system has not yet fully matured, rendering humoral immunity particularly vulnerable [35]. Secretory immunoglobulin A (sIgA) is produced by plasma cells in the intestinal lamina propria and is regulated by T cell cytokines. Th2-type cytokines (e.g., IL-4, IL-5, IL-6, IL-10) upregulate IgA production, whereas Th1-type cytokines (e.g., IFN-γ, TNF-β) exert inhibitory effects, and weaning stress may disrupt this balance and impair stable sIgA secretion [35]. Psychological and social stressors, dietary changes, and potential intestinal barrier damage during weaning can interfere with normal B-cell activation and antibody production pathways, resulting in reduced systemic and mucosal humoral immune responses, including serum IgG, IgA, and IgM, and consequently increasing susceptibility to pathogens such as ETEC.

During infection, pathogen-associated molecular patterns such as lipopolysaccharide and flagellin are recognized by host pattern recognition receptors, activating NF-κB and MAPK signaling and driving robust production of pro-inflammatory mediators [36]. TLR4-related cytokines and chemokines, including IL-8 and CXCL5 that recruit neutrophils, can promote excessive immune cell infiltration and exacerbate intestinal tissue injury when insufficiently controlled [37,38]. If anti-inflammatory regulation such as IL-10 is relatively insufficient, inflammation spreads and accelerates barrier collapse, ultimately contributing to diarrhea.

## 3. Plant Extracts in the Alleviation of PWD

Plant extracts and their by-products are rich in diverse bioactive phytochemicals—including polysaccharides, polyphenols, essential oils, alkaloids and other secondary metabolites—which contribute to their biological activities and potential applications in animal health and nutrition [39]. These compounds have demonstrated broad potential applications in the medical, food, and agricultural industries [40,41,42] and possess multiple advantages, such as natural origin, multifunctional activity, and a low propensity to induce antimicrobial resistance [43]. Plant extracts have been confirmed to alleviate PWD through multiple pathways. In this section, several representative categories of plant extracts are selected to elucidate their mechanisms of action against PWD, thereby exploring the potential of plant-derived additives as novel feed additives for the prevention and control of PWD. In this review, “plant extracts” refers specifically to phytogenic (plant-derived) extracts and does not include organic-acid acidifiers that may be synthetically produced (e.g., benzoic acid).

For clarity, plant extracts are discussed according to their primary regulatory roles, including modulation of feed intake, immune and inflammatory responses, oxi-dative stress, intestinal barrier integrity, and gut microbiota composition. Where available, we emphasize functional outcomes and intervention evidence when discussing mechanisms; otherwise, changes in microbiota, metabolites, or gene expression are interpreted as supportive clues rather than definitive proof. Given the bidirectional relationship between dysbiosis and diarrhea discussed in Section 2.3, microbiota-related outcomes are interpreted alongside pathogen- and host-directed evidence to distinguish causal mediators of diarrhea reduction from secondary markers of gut recovery.

### 3.1. Polysaccharides-Based Plant Extracts

Plant-derived polysaccharides originate from the long history of herbal medicine application and are widely used due to their positive effects on health. In both traditional herbal medicine and modern nutritional intervention studies, polysaccharides are often regarded as functional components with holistic regulatory properties, and their effects are not limited to a single target. Studies have demonstrated that polysaccharides can enhance the resistance of weaned piglets through multiple pathways, including increasing feed intake, enhancing immune function and regulating inflammation, and modulating the gut microbiota, thereby effectively improving their health status and exhibiting comprehensive advantages in coping with the complex pathological process of weaning stress, which involves multiple interacting factors.

Feed is the primary source of energy to meet the growth requirements of animals [44], and improving feed intake is key for weaned piglets to overcome growth stagnation and ensure nutrient intake. Multiple polysaccharides have been proven to effectively promote this process. Research indicates that dietary supplementation of *Lycium barbarum* polysaccharide (LBP) significantly increases the ADFI of weaned piglets [15]. Supplementation with *Laminaria japonica* polysaccharide (LJP) shows similar effects, with the addition of 200 and 400 mg/kg LJP linearly increasing ADG and ADFI [45]. Although mulberry (*Morus alba* L.) leaf polysaccharides (MLPs) do not significantly affect ADG or feed conversion rate, they can increase the feed intake of piglets [46]. These findings indicate that the feed intake–promoting effects of polysaccharides exhibit a certain degree of commonality among different sources and structural types, whereas their impacts on growth performance indices may vary. The improvement in feed intake may be partly attributed to the overall enhancement of intestinal health by polysaccharides, including improved digestive function and maintenance of intestinal structure, thereby stimulating appetite and feeding behavior. Adequate nutrient intake provides the material basis for effective immune system function and tissue repair, serving as a necessary condition for resisting diarrhea and as an important prerequisite for the subsequent immunomodulatory and microecological regulatory effects of polysaccharides.

One reason for PWD is reduced immune function, and enhancing the body’s immune capacity is crucial for preventing and alleviating diarrhea. Polysaccharides can regulate the immune system of weaned piglets through multiple pathways, primarily by enhancing humoral immunity, cellular immunity, and modulating inflammatory responses, thereby alleviating immune imbalance caused by weaning stress and pathogen infection. In terms of humoral immunity, dietary supplementation with *Lycium barbarum* polysaccharide (LBP) and *Achyranthes bidentata* polysaccharide (ABP) significantly increases serum levels of IgG and IgM [15,47]. Research on Isatis root polysaccharide (IRP) further confirms that as the supplementation concentration increases, serum IgA and IgG levels show a significant upward trend, and IgM levels also increase linearly [48]. In terms of cellular and humoral immune regulation relevant to PWD, a polysaccharide-rich *Codonopsis pilosulae* and *Astragalus membranaceus* extract (CA) reduced diarrhea rate in 31-day-old weaned piglets and improved systemic immune indices, including serum immunoglobulins. This supports the use of plant polysaccharide-based interventions to alleviate PWD partly through enhanced immune competence [49]. Additionally, polysaccharides exhibit significant anti-inflammatory activity. IRP increases the gene expression of the anti-inflammatory factor IL-10 in the jejunum and ileum while reducing the expression of the pro-inflammatory factor IL-1β [48]. Together, these immunomodulatory and anti-inflammatory effects form an important foundation for defending against pathogens and maintaining intestinal immune homeostasis, providing immunological support for reducing the risk of infectious diarrhea during the weaning period.

As important prebiotics, polysaccharides can precisely regulate the intestinal microbial community, thereby improving weaning diarrhea in piglets caused by dysbiosis at the microecological level. Because polysaccharides are difficult to degrade by host digestive enzymes, they can serve as fermentable substrates for intestinal microorganisms and selectively promote the growth of beneficial bacteria. Supplementation with *Astragalus* polysaccharide (APS) and *Ginseng* polysaccharide (GPS) can enhance the species richness and evenness of colonic bacteria, increasing the relative abundance of beneficial bacteria such as *Lachnospiraceae* and *Anaerostipes* [50]. Meanwhile, MLPs inhibit intestinal *Escherichia coli* while effectively promoting the growth of *Lactobacillus* and *Bifidobacterium* [46]. Similarly, dietary supplementation with 0.20% fermented purslane (*Portulaca oleracea* L.), a polysaccharide-rich edible medicinal herb, reduced diarrhea incidence in weaned piglets and was accompanied by increased fecal butyrate, supporting a potential microbiota–metabolite route for gut health benefits [51]. Mechanistically, polysaccharides can be framed as fermentable substrates that escape host digestion and are utilized by gut commensals, thereby increasing fermentation products such as SCFAs [50]. Elevated SCFAs may reinforce epithelial barrier integrity and immune homeostasis [48], while also constraining pathogen expansion by improving colonization resistance and lowering luminal pH [52]. Thus, microbiota shifts after polysaccharide supplementation may represent candidate mediators of diarrhea reduction, but they may also be secondary correlates; this hypothesis can be tested by coupling SCFA quantification with barrier/immune readouts and causal validation (e.g., microbiota depletion/FMT or SCFA add-back).

In summary, polysaccharide extracts construct a comprehensive and multi-layered host endogenous defense network through the synergistic actions of immunomodulation and anti-inflammation, increased feed intake, and optimization of the gut microbiota, thereby effectively combating PWD in piglets. This multi-target and systemic regulatory strategy confers unique advantages to polysaccharides in addressing the complex pathological changes occurring during the weaning period. It is worth noting that although some studies suggest that polysaccharides can enhance antioxidant enzyme activity and reduce oxidative damage markers [15], compared with the substantial evidence supporting their roles in immune regulation and microbiota modulation, studies on the mechanisms by which polysaccharides alleviate PWD in piglets through direct antioxidant pathways remain relatively limited, and the antioxidant efficacy and signaling pathways of different polysaccharides still require further systematic investigation. The effects and mechanisms of plant-derived polysaccharides in alleviating PWD are summarized in Table 1.

**Table 1:** Effects and actions of plant-derived polysaccharides in alleviating PWD in piglets.

No.	Polysaccharide	Dose and Delivery	Model	Sample Size and Duration	Main Outcomes	Mechanistic Evidence	Reference
1	*Lycium barbarum* polysaccharides (LBPs)	Trial 1: 1000, 2000, 4000, 6000 mg/kg in feed; Trial 2: 4000 mg/kg in feed	Weaned piglets (Yorkshire × Landrace × Duroc)	Trial 1: *n* = 400, 14 d; Trial 2: n = 32, 14 d	↑ ADG, ↑ ADFI; ↓ diarrhea incidence	↑ serum IgG, IgM; ↑ SOD, CAT, GSH-Px; ↓ MDA; ↑ *Bacteroidetes*, *Lactobacillus*, *Bifidobacterium*; ↓ *E. coli*	[15]
2	*Laminaria japonica* polysaccharide (LJP)	100, 200, 400 mg/kg in feed	Weaned piglets (Barkshire × Licha Black)	*n* = 120; 21 d	↑ ADG, ↑ ADFI; ↑ fecal amylase and lipase activity	↑ digestive enzyme activity; ↑ serum histidine and asparagine	[45]
3	Mulberry leaf polysaccharides (MLPs)	0.3, 0.6, 0.9 g/kg in feed	weaned piglets (Duroc–Landrace–Yorkshire)	*n* = 150; 21 d	↓ diarrhea incidence; improved feed efficiency	↓ *Escherichia coli*; ↑ *Lactobacillus and Bifidobacterium*; ↑ IGF-1, GH	[46]
4	*Achyranthes bidentata* polysaccharides (ABPS)	500 mg/kg in feed	Weaned piglets (Landrace × Large White)	*n* = 120; 28 d	↑ ADG; ↓ fecal score	↑ IgA, IL-2; ↓ IL-1β; ↑ ileal VH, jejunal sIgA	[47]
5	Isatidis root polysaccharide (IRP)	0.1%, 0.2%, 0.5%, 1.0% in feed	Weanling piglets (Duroc × Landrace × Yorkshire)	*n* = 40; 21 d	↓ diarrhea score	↑ serum IgA, IgG, IgM; ↑ ZO-1, occludin, MUC2; ↑ IL-10; ↓ *E. coli*	[48]
6	*Codonopsis pilosula*+ *Astragalus membranaceus* (CA)	0.5%, 1.0%, 1.5% in feed	Weaned piglets (Duroc × Landrace × Large White)	*n* = 48, 28 d	↑ ADG, ↑ immune function, ↓ diarrhea rate	↑ SCFAs, *Firmicutes*, *Lactobacillus* abundance	[49]
7	*Astragalus* polysaccharide (Aps)/*Ginseng* polysaccharide (Gps)	800 mg/kg in feed	Weaned piglets (Duroc × Landrace × Yorkshire)	*n* = 180; 28 d	↑ADG; ↓ diarrhea rate	↑ TLR4, MyD88, NF-κB; ↑ SCFAs; ↑ *Lachnospiraceae*, *Anaerostipes*	[50]
8	Fermented Purslane (FP)	0.20% in feed	Weaned piglets (Duroc × (Landrace × Large White))	*n* = 48, 28 d	↑ ADG, decreased diarrhea, improved immune function	↑ SOD, ↓ CAT, reduced IL-6, enhanced microbial butyrate metabolism	[51]

Abbreviations: PWD, post-weaning diarrhea; ADG, average daily gain; ADFI, average daily feed intake; IgA/IgG/IgM, immunoglobulin A/G/M; SOD, superoxide dismutase; CAT, catalase; GSH-Px, glutathione peroxidase; MDA, malondialdehyde; SCFAs, short-chain fatty acids; TLR4, Toll-like receptor 4; MyD88, myeloid differentiation primary response 88; NF-κB, nuclear factor kappa B; VH, villus height; ZO-1, zonula occludens-1; MUC2, mucin 2; sIgA, secretory immunoglobulin A; IGF-1, insulin-like growth factor 1; GH, growth hormone; IL, interleukin; LBPs, *Lycium barbarum* polysaccharides; LJP, *Laminaria japonica* polysaccharide; MLPs, mulberry leaf polysaccharides; ABPS, *Achyranthes bidentata* polysaccharides; IRP, Isatidis root polysaccharide; ↑, increased; ↓, decreased.

### 3.2. Polyphenols-Based Plant Extracts

Polyphenols are a class of secondary metabolites derived from plants, whose chemical structure is characterized by one or more aromatic rings bearing hydroxyl groups [53]. According to structural differences, polyphenolic compounds can be further classified into several subclasses, including flavonoids, phenolic acids, tannins, and lignans, with different structural types exhibiting distinct biological functions. Extensive research has demonstrated that polyphenols possess multiple biological activities, including anti-inflammatory, antibacterial, antioxidant effects, and the ability to improve intestinal permeability [54], conferring them significant research value in the regulation of intestinal health and the prevention and control of stress-related diseases.

Excessive ROS induced by weaning is a key factor in causing intestinal damage [31]. Polyphenols can directly act as electron donors, scavenging ROS and terminating the oxidative chain reaction [55], thus reducing oxidative damage to cellular membranes, proteins, and nucleic acids. Papakonstantinou et al. demonstrated that adding tannic acid to the diet significantly reduced the MDA content in the ileum of weaned piglets, supporting the antioxidant potential of polyphenols in mitigating local lipid peroxidation damage in the intestine [56]. Furthermore, polyphenols may modulate antioxidant-related signaling pathways, and some studies report increased expression or activity of endogenous antioxidant enzymes, which is consistent with an enhanced systemic antioxidant defense. Liu et al. showed that gardenia fruit powder significantly increased the activities of T-AOC, T-SOD and CAT in the serum and intestine of weaned pigs [57]. More in-depth mechanistic studies have shown that flavonoid quercetin can activate the Nrf2/Keap1 signaling pathway, promoting the expression of various downstream antioxidant genes, thus systemically enhancing the body’s ability to respond to oxidative stress [58].

Oxidative stress is closely linked to inflammatory responses. In addition to scavenging ROS, polyphenols play important roles in anti-inflammatory and immune regulation. Licorice flavonoid powder significantly downregulated the mRNA expression of pro-inflammatory cytokines IL-1β and IL-8 in the duodenum, while upregulating the expression of IL-10 in the ileum, suggesting modulation of inflammation [59], which may contribute to a reduced intestinal mucosal inflammatory response induced by weaning stress. Chlorogenic acid has been shown to inhibit the TLR4/NF-κB inflammatory signaling pathway and was associated with reduced concentrations of IL-1β, IL-6, and TNF-α in both serum and the intestine, which may help alleviate intestinal inflammation [60]. These anti-inflammatory effects complement the antioxidant properties, working together to reduce the damage to the intestinal mucosa caused by weaning stress.

Numerous studies have shown that polyphenols have positive effects on improving intestinal morphology and structure. Dietary supplementation with gardenia fruit powder significantly increased the villus height (VH) and the villus height/crypt depth (VH/CD) ratio in the ileum of weaned piglets, suggesting its role in enhancing the intestinal digestive and absorption surface area [57]. Similarly, licorice flavonoid powder has been found to increase villus height and the VH/CD ratio in the duodenum [59]. The addition of grapefruit peel powder also increased villus height in the ileum and reduced the incidence of diarrhea [61]. At the same time, polyphenols were associated with increased expression or abundance of tight junction proteins, which is consistent with improved epithelial barrier status. Chlorogenic acid supplementation significantly increased the protein abundance of Claudin 1, Occludin, and ZO-1 in the jejunum and ileum of weaned pigs [60]. Tannic acid can upregulate the mRNA expression of Occludin and ZO-2 in the duodenum. Tea residue extract, although not significantly affecting related gene expression, can directly inhibit the activity of TMEM16A and CFTR ion channels in intestinal epithelial cells (HT-29), helping maintain intestinal homeostasis by reducing excessive intestinal fluid secretion [62]. This mechanism provides a new explanation for alleviating diarrhea from the perspective of secretion regulation. These improvements may contribute to improved nutrient absorption capacity and reduce the abnormal fermentation of undigested nutrients in the hindgut, potentially lowering the risk of diarrhea. Collectively, these findings support a host-directed mechanism whereby polyphenols alleviate PWD by strengthening epithelial barrier integrity and limiting excessive fluid secretion.

In addition to modulating host responses, polyphenols also exert pathogen-directed actions that can directly interrupt key steps in ETEC-associated diarrhea. Both in vitro and in vivo studies have confirmed that certain polyphenolic extracts (e.g., Omnivin and ALSOK) can inactivate heat-labile toxins (LT) produced by ETEC, and can also inhibit ETEC adhesion to epithelial receptors or reduce early-stage colonization by inducing bacterial aggregation [63]. Tannic acid may further suppress *E. coli* growth by chelating iron and depriving pathogens of essential nutrients [16].

Several studies additionally report shifts in gut microbiota composition following polyphenol supplementation, often characterized by enrichment of putative beneficial genera such as *Lactobacillus* and *Bifidobacterium* [60]. For example, a weaned-piglet study of dietary silybin reported a shift in gut microbiota toward short-chain fatty-acid-producing taxa, alongside improved gut health indices [64]. However, because microbiota endpoints are typically measured concurrently with or after clinical improvement and reduced pathogen burden, it remains unclear whether these microbiota changes mediate diarrhea reduction or primarily reflect recovery of a healthier gut environment.

Overall, polyphenols likely alleviate PWD through complementary host-directed and pathogen-directed mechanisms, including reinforcement of barrier function, control of epithelial secretion, and antitoxin, anti-adhesion, and growth-inhibitory effects. Microbiota modulation may contribute in some contexts, but current evidence in weaned piglets is largely associative; causal validation is still needed to determine whether microbiota shifts drive efficacy or instead reflect gut recovery. Key studies on polyphenol-based plant extracts and their reported effects on PWD are summarized in Table 2.

**Table 2:** Effects and actions of plant-derived polyphenols in alleviating PWD in piglets.

No.	Polyphenols	Dose and Delivery	Model	Sample Size and Duration	Main Outcomes	Mechanistic Evidence	Reference
1	Tannin blend (TAN)	0.25 g/kg in feed	Weaned piglets (Duroc × Landrace × Yorkshire)	*n* = 81; 30 d	↓ PWD; ↑ ADG	↓ diarrhea odds; effect associated with higher initial body weight	[56]
2	Grape extract (GE)	0.4%, 0.6%,0.8% in feed	Weaned piglets (Duroc × (Landrace × Yorkshire))	*n* = 144; 21 d	↓ F/G and diarrhea rate	↑ antioxidant capacity (T-AOC, SOD, CAT, GSH-Px); ↑ villus height and VH/CD; ↑ ZO-1, Occludin; activation of Nrf2 pathway; altered gut microbiota	[57]
3	Quercetin	0.2% in feed	ETEC (K88)-challenged weaned piglets (Duroc × Landrace × Yorkshire)	*n* = 24: 24 d	↓ inflammation and oxidative stress; ↓ diarrhea	↑ IgA, IgM, IgG; ↑ SOD, CAT, GSH-Px; activation of Nrf2/HO-1; ↓ QS signaling and *E. coli*	[58]
4	Licorice flavonoids powder (LFP)	50, 150, 250 mg/kg in feed	Weaned piglets (Duroc × Landrace × Yorkshire)	*n* = 96; 35 d	↓ diarrhea index; improved growth trend	↑ villus height; ↑ OCLN expression; ↓ IL-1β, IL-8, iNOS; ↑ IL-10	[59]
5	Chlorogenic acid (CGA)	200 mg/kg in feed	Weaned piglets under oxidative stress (Duroc × Landrace × Yorkshire)	*n* = 24; 21 d	↓ diarrhea rate; improved intestinal morphology	↑ ZO-1, occludin, claudin-1; ↑ Lactobacillus; inhibition of TLR4/NF-κB; activation of Nrf2	[60]
6	Pomelo peel powder (PPP)	8 g/kg in feed	Weaned piglets (Duroc × Landrace × Yorkshire)	*n* = 30; 28 d	↑ ADG, ↑ ADFI; ↓ diarrhea	↑ antioxidant enzymes; ↑ IgA, IgG; altered colonic microbiota composition	[61]
7	Tea residue	1 kg/t in feed	Weaned piglets (Duroc × Landrace × Yorkshire)	*n* = 36; 28 d	↓ diarrhea rate	Inhibition of TMEM16A and CFTR chloride channels; ↓ fecal Cl^−^ secretion	[62]
8	Plant polyphenol extracts (Omnivin, ALSOK)	1% in feed	ETEC-infected weaned piglets (not mention)	*n* = 40; 16 d	↓ ETEC-induced diarrhea	Inactivation of ETEC heat-labile toxin (LT) in vivo; ↓ ETEC excretion	[63]
9	Silybin	50, 100, 200, 400 mg/kg in feed	weaned piglets (Duroc × Landrace × Yorkshire)	*n* = 120, 42 d	↓ Diarrhea, ↑ Antioxidant capacity; diarrhea	↑ Nrf2, SOD; ↓ MDA, IL-1β	[64]

Abbreviations: PWD, post-weaning diarrhea; ADG, average daily gain; ADFI, average daily feed intake; F/G, feed-to-gain ratio; T-AOC, total antioxidant capacity; SOD, superoxide dismutase; CAT, catalase; GSH-Px, glutathione peroxidase; VH, villus height; CD, crypt depth; VH/CD, villus height-to-crypt depth ratio; ZO-1, zonula occludens-1; OCLN (Occludin), occludin; iNOS, inducible nitric oxide synthase; Nrf2, nuclear factor erythroid 2–related factor 2; HO-1, heme oxygenase-1; TLR4, Toll-like receptor 4; NF-κB, nuclear factor kappa B; ETEC, enterotoxigenic *Escherichia coli*; QS, quorum sensing; TMEM16A, anoctamin 1; CFTR, cystic fibrosis transmembrane conductance regulator; TAN, tannin blend; GE, grape extract; LFP, licorice flavonoids powder; CGA, chlorogenic acid; PPP, pomelo peel powder; ↑, increased; ↓, decreased.

### 3.3. Essential Oils-Based Plant Extracts

Volatile aromatic compound extracts obtained by distillation from natural plants are referred to as essential oils [65]. Essential oils possess antibacterial, anti-inflammatory, and antioxidant properties and are commonly used as edible additives. Their major active components include terpenoids, phenolic compounds, and their derivatives, which confer essential oils with broad and stable biological functions. Numerous experimental studies have demonstrated that essential oils exert regulatory effects on PWD and exhibit favorable application potential in alleviating weaning stress–related injuries.

Essential oils can effectively enhance the antioxidant defense system of weaned piglets, which constitutes the primary line of defense against oxidative stress and the maintenance of intestinal cellular homeostasis. Studies have shown that supplementation with tea tree oil (TTO) significantly increases serum activities of SOD, GSH-Px, and total antioxidant capacity (T-AOC) in weaned piglets while reducing the levels of the lipid peroxidation product MDA, suggesting improved enzymatic and non-enzymatic antioxidant status [66,67]. Additionally, TTO treated with encapsulation technology better preserves its active components, thereby more effectively elevating serum SOD and GSH-Px levels [67]. Furthermore, rice bran oil (RBO) has also been confirmed to alleviate LPS-induced oxidative stress, increase plasma activities of CAT and SOD as well as total antioxidant capacity, and reduce MDA concentration [68]. By neutralizing excessive reactive oxygen species and attenuating lipid peroxidation damage, essential oils may help maintain the integrity and function of intestinal epithelial cells, potentially reducing secondary oxidative damage to intestinal barrier structures.

While alleviating oxidative damage, essential oils modulate the host’s immune response through multiple pathways to balance inflammation and enhance specific immunity. Serum immunoglobulins are generally regarded as indicators of humoral immunity in animals. Dietary supplementation with essential oils (such as carvacrol and thymol) combined with probiotics significantly increases serum IgG concentrations in weaned piglets [69], suggesting that essential oils can promote the recovery of humoral immune function to a certain extent. In addition to modulating immunoglobulins, essential oils and related substances can finely regulate the inflammatory cytokine network. Research by Pu et al. demonstrated that oregano oil, when co-supplemented with benzoic acid (an organic-acid acidifier that is not necessarily plant-derived), reduced serum TNF-α concentrations and lowered serum IL-1β levels. Therefore, these outcomes should be interpreted as evidence from a combination-additive strategy rather than an essential-oil-only effect [70].

One of the core advantages of essential-oil-based substances is their ability to suppress enteric pathogens and thereby reduce diarrhea. In vitro, citrus essential oil increases membrane permeability and disrupts the integrity of ETEC cells, indicating a direct antimicrobial effect [71]. Consistently, in weaned piglets, encapsulated tea tree oil (TTO) markedly reduced the abundance of *Escherichia–Shigella* in the cecum and colon, and its diarrhea-reducing efficacy was comparable to that of antibiotics [67]. Together, these findings support a pathogen-directed chain from antimicrobial activity to reduced intestinal pathogen colonization and, consequently, lower diarrhea incidence.

Beyond pathogen suppression, essential oils may also contribute through a microbiota–metabolite route. TTO increased the proportions of Firmicutes and Bacteroidetes and enriched butyrate-producing bacteria as well as SCFA-producing Clostridium species [67]. Importantly, essential oil supplementation has been associated with increased concentrations of fermentation products, including propionate and butyrate [66]. These metabolite shifts provide functional evidence that microbiota changes are accompanied by altered microbial outputs, which may support barrier integrity and immune homeostasis. Nevertheless, because microbiota and SCFAs are often measured concurrently with clinical improvement, further causal validation is needed to determine whether these changes mediate diarrhea reduction or reflect recovery of gut health.

In summary, essential oils construct a multi-layered and three-dimensional host defense system through the synergistic and integrated actions of antioxidation, immune enhancement, and microbiota modulation, contributing to the prevention and control of PWD in piglets. Notably, evidence for essential oils includes both pathogen-directed suppression (reduced intestinal pathogen abundance) and microbiota–metabolite signals (elevated SCFAs), suggesting multiple concurrent routes of action. This multi-target and comprehensive mode of action confers unique advantages to essential oils in addressing the complex pathological changes occurring during the weaning period. Essential oils, particularly products with improved stability and bioavailability through techniques such as encapsulation, represent effective strategies for replacing in-feed antibiotics and safeguarding intestinal health by comprehensively enhancing the endogenous defense capacity of weaned piglets. The main evidence for essential oils in alleviating PWD, including performance and mechanistic readouts, is summarized in Table 3.

**Table 3:** Effects and actions of essential oils and combination strategy in alleviating PWD in piglets.

No.	Essential Oils	Dose and Delivery	Model	Sample Size and Duration	Main Outcomes	Mechanistic Evidence	Reference
1	Tea tree oil (TTO)	100 mg/kg in feed	Weaned pigs (Duroc × Landrace × Yorkshire)	*n* = 216; 28 d	↑ Gain to feed ratio; ↓ diarrhea incidence	Improved nutrient digestibility; ↑ SOD, IL-10; ↑ propionate and butyrate; altered gut microbiota	[66]
2	Tea tree oil (TTO)	0.4% in feed	Weaned pigs (Duroc × Landrace × Large white)	*n* = 144; 28 d	↑ ADG, ↑ ADFI; ↓ diarrhea	↑ antioxidant capacity; ↑ IgG; ↑ abundance of *Clostridium_sensu_stricto_1*; ↓ *Escherichia-Shigella*	[67]
3	Rice bran oil (RBO)	0.01%, 0.02%, 0.03% in feed	Weaned piglets (Landrace × Yorkshire)	*n* = 168; 21 d	↑ ADG, ↑ ADFI; ↓ diarrhea	Improved jejunal villus height, reduced apoptosis; ↑ catalase, SOD, IgA, IgM	[68]
4	Essential oils (thymol, carvacrol) + Bacillus probiotic (B. subtilis; combination strategy)	300, 600, 1000 g/tonne in feed	Weaned pigs (Yorkshire × Landrace × Duroc)	*n* = 96; 28 d	↑ ADG; ↓ diarrhea incidence	↑ serum IgG; ↓ ammonia emission; altered gut microbiota	[69]
6	Oregano oil (AO) + benzoic acid( organic-acid acidifier; combination strategy)	400 g/t + 3000 g/t infeed	Weaned piglets (Duroc × [Landrace × Yorkshire])	*n* = 25; 26 d	↓ serum TNF-α; ↑ immune status;	↓ inflammatory factors	[70]

Abbreviations: ADG, average daily gain; ADFI, average daily feed intake; SOD, superoxide dismutase; IgG, immunoglobulin G; IL-10, interleukin 10; TTO, tea tree oil; RBO, rice bran oil; AO, Oregano oil; ↑, increased; ↓, decreased.

### 3.4. Alkaloids-Based Plant Extracts

Alkaloids are secondary compounds mainly discovered in plants and are nitrogen-containing natural compounds. Alkaloid-containing plants have been used for pharmaceutical purposes since ancient times [72]. From the perspectives of chemical structure and biological activity, alkaloids exhibit a high degree of diversity and constitute an important component of plant defense systems. Various alkaloids have been discovered to possess antioxidant, anti-inflammatory, antibacterial, and analgesic effects, and have been clinically applied in the treatment of numerous diseases such as Alzheimer’s disease, cancer, and hypertension [73,74,75]. These studies provide an important theoretical basis for the application of alkaloids in the field of animal health. Multiple studies have demonstrated that alkaloids exhibit favorable therapeutic effects on diarrhea caused by ETEC in weaned piglets.

Alkaloid-based substances can effectively alleviate oxidative damage in weaned piglets induced by environmental stress and pathogen infection. Wang et al. found that berberine (BBR), in a weaned piglet model, associated with alleviate intestinal oxidative damage induced by ETEC infection, with regulation of the Nrf2 signaling pathway accompanied by increased transcription of intracellular antioxidant defense components [76]. Supplementation with Macleaya cordata extract (MCE) associated with increased activities of host antioxidant enzyme system. Studies have shown that piglets fed MCE exhibited significantly increased serum total antioxidant capacity (T-AOC), glutathione peroxidase (GSH-Px), and superoxide dismutase (SOD) activities, while malondialdehyde (MDA) content was reduced [77]. Similarly, Piper sarmentosum extract (PSE) has also been found to increase serum GSH-Px activity and decrease MDA levels in weaned piglets, suggesting antioxidant effects [78]. These results indicate that alkaloids from different sources can synergistically attenuate oxidative stress–induced damage through multiple mechanisms, and these antioxidant effects create a favorable intracellular environment for subsequent barrier repair and inflammation control.

While alleviating oxidative stress, alkaloid-based substances may act on intestinal structures to strengthen physical barrier function. Berberine is particularly notable in this regard. Research shows that berberine supplementation significantly alleviates intestinal mucosal damage caused by ETEC infection, specifically manifested as increased jejunal villus height and villus-to-crypt ratio, as well as increased mucosal thickness in the duodenum, jejunum, and ileum [76]. Proposed mechanisms include upregulation of genes related to tight junctions and adherens junctions, which is consistent with enhanced epithelial cell connectivity and reduced intestinal permeability. However, low doses of berberine (0.25–0.5 g/kg) can increase ileal villus height and villus-to-crypt ratio, while excessively high doses (1 g/kg) may produce opposite effects, indicating that its regulation of intestinal morphology is dose-dependent [79]. Strengthening the physical barrier may help prevent the translocation of pathogens and toxins.

Notably, evidence from berberine studies illustrates the challenge of interpreting “microbiota modulation” as a causal mechanism. In weaned piglets receiving a low dose of berberine at 30 mg/kg in feed, no significant improvement in growth or diarrhea was observed, and this null efficacy coincided with no detectable changes in gut morphology, gut microbiota, or SCFAs, despite the presence of phase I/II metabolites [79]. By contrast, in an ETEC-challenged piglet model, dietary berberine at 0.05% or 0.1% improved intestinal morphology and barrier-related indices, reduced inflammatory responses, and was accompanied by increased SCFAs and a reshaped microbiota [80]. Together, these divergent findings are consistent with, but do not prove, the possibility that engaging the microbiota–metabolite axis may be linked to therapeutic efficacy under certain doses, formulations, or disease contexts. Future work should therefore incorporate causal validation, such as microbiota depletion followed by fecal microbiota transplantation and metabolite-focused testing including SCFA add-back, to determine whether microbiota changes mediate diarrhea reduction or simply reflect recovery of gut homeostasis.

In summary, alkaloid-based substances construct a multidimensional host defense network through three interrelated and synergistic pathways—antioxidant activity, barrier repair, and microbiota modulation—thereby effectively combating diarrhea in weaned piglets. Consequently, alkaloid-based substances demonstrate significant potential as substitutes for feed antibiotics, with the core rationale lying in their multi-target intervention to comprehensively enhance the endogenous defense capacity of the intestines in weaned piglets. Nevertheless, efficacy may vary across doses and disease contexts, and microbiota-related findings should be interpreted in light of causal evidence. Representative alkaloid-based interventions, their doses, models, and outcomes related to diarrhea and gut health are summarized in Table 4.

**Table 4:** Effects and actions of alkaloid-based substances in alleviating PWD in piglets.

No.	Alkaloid	Dose and Delivery	Model	Sample Size and Duration	Main Outcomes	Mechanistic Evidence	Reference
1	Berberine	0.05%, 0.1% in feed	ETEC-challenged weaned piglets (Duroc × Landrace × Large Yorkshire)	*n* = 24; 21 d	↑ BW, ADG, ADFI; ↓ diarrhea incidence	↓ TNF-α, IL-1β, IL-6, IL-8; inhibition of TLR4/MyD88/NF-κB; activation of Nrf2 pathway; ↑ beneficial bacteria, ↓ pathogenic bacteria	[76]
2	Macleaya cordata extract (MCE)	50 mg/kg in feed	Weaned piglets (Duroc × (Large White × Landrace))	*n* = 36; 21 d	↑ ADG; ↓ feed efficiency and diarrhea rate	↑ serum IgG; ↑ T-AOC, GSH-Px, SOD; ↑ villus height and VH/CD; ↑ *Lactobacillus*, ↓ *Salmonella* and *E. coli*	[77]
3	Piper sarmentosum e xtract (PSE)	50, 100, 200 mg/kg in feed	Weaned piglets (Duroc × Landrace × Yorkshire)	*n* = 80; 28 d	↑ ADG and ADFI	↑ GSH-Px; ↓ MDA; ↓ IL-1β, TNF-α, IL-6; ↑ IL-4, IL-10, TGF-β	[79]
4	Berberine (BBR)	30 mg/kg in feed	Weaned piglets (TN70 × Piétrain)	*n* = 60; 14 d	No significant effects on growth or diarrhea	Berberine metabolized to phase I/II metabolites; no significant changes in gut morphology, microbiota, or SCFAs	[79]
5	Berberine	0.05% or 0.1% in feed	ETEC-challenged weaned piglets (Duroc × Landrace × Large Yorkshire)	*n* = 24; 21 d	Improved intestinal morphology	↑ villus height, VH/CD, goblet cells; ↑ ZO-1, ZO-2, Claudin-1, Occludin; ↓ apoptosis; ↓ pro-inflammatory cytokines; ↑ SCFAs; reshaped microbiota	[80]

Abbreviations: BW, body weight; ADG, average daily gain; ADFI, average daily feed intake; IgG, immunoglobulin G; T-AOC, total antioxidant capacity; SOD, superoxide dismutase; GSH-Px, glutathione peroxidase; MDA, malondialdehyde; VH, villus height; CD, crypt depth; VH/CD, villus height-to-crypt depth ratio; IL, interleukin; TNF-α, tumor necrosis factor alpha; TGF-β, transforming growth factor beta; TLR4, Toll-like receptor 4; MyD88, myeloid differentiation primary response 88; NF-κB, nuclear factor kappa B; SCFAs, short-chain fatty acids; ZO-1/ZO-2, zonula occludens-1/2; ETEC, enterotoxigenic *Escherichia coli*; BBR, berberine; MCE, Macleaya cordata extract; PSE, Piper sarmentosum extract; ↑, increased; ↓, decreased.

## 4. Challenges, Limitations and Future Directions

### 4.1. Evidence Quality and Causal Validation (From Efficacy to Confidence)

A central limitation in the current literature is mechanistic attribution. Across extract classes, many studies report concurrent improvements in diarrhea outcomes together with shifts in gut microbiota profiles and/or SCFAs, yet it often remains unclear whether these changes causally drive clinical improvement or simply accompany the restoration of gut homeostasis. Future research should therefore strengthen causal inference by integrating pathogen- and toxin-focused endpoints (e.g., ETEC colonization, toxin activity, and virulence indicators) with host functional readouts (intestinal barrier integrity, ion transport, and inflammatory signaling), rather than relying primarily on associative biomarkers. When a microbiota-mediated mechanism is proposed, experimental designs that directly test causality—such as microbiota depletion followed by fecal microbiota transplantation (FMT)—will be particularly informative, as exemplified by capsule-based FMT alleviating PWD in piglets through microbiota modulation [28]. At the same time, methodological rigor is essential because FMT-based causality testing also has recognized pitfalls and confounders (e.g., donor effects, housing effects, and incomplete microbiota transfer) [27]. Complementary “metabolite mediation” strategies may also help clarify whether SCFAs are drivers or bystanders, since direct SCFA infusion has been shown to improve intestinal barrier function in weaned piglets [29]. Collectively, these approaches would help identify the dominant drivers of benefit across phytochemical categories and enable more precise, evidence-based formulation. Importantly, mechanistic confidence should be developed in parallel with a clearer, extract-specific safety framework before positioning plant extracts as replacements for antibiotics or pharmacological ZnO.

Importantly, mechanistic confidence should be developed in parallel with a clearer, extract-specific safety framework before positioning plant extracts as replacements for antibiotics or pharmacological ZnO. In parallel, clinical confidence would benefit from more standardized reporting of diarrhea endpoints and effect magnitude, because baseline diarrhea burden and outcome definitions vary substantially across studies. Diarrhea outcomes are variably presented as incidence, diarrhea rate, or fecal scores, often over non-overlapping post-weaning windows, which complicates quantitative comparisons between trials. In addition, differences in weaning age, basal diet formulation, and pathogen pressure (natural exposure during the nursery period versus experimental challenge) likely contribute to both the starting level of diarrhea and the apparent magnitude of response. Where baseline and endpoint values are reported, the effect size appears context-dependent. For instance, tea tree oil supplementation reduced diarrhea incidence during d 0–14 from 9.23% in the control group to 5.48% (−3.75 percentage points; ~40.6% relative reduction) [66]. In piglets with higher baseline diarrhea later in the nursery period, pomelo peel powder reduced diarrhea rate from 36.6% to 10.7% in week 3 (−25.9 points; ~70.8% relative reduction) and remained lower in week 4 (35.6% to 14.4%) [61]. Under an ETEC challenge model, berberine decreased diarrhea rate from 7.33% to 2.61% (0.05% berberine) and 2.38% (0.1% berberine), corresponding to ~64–68% relative reductions [76]. However, not all trials provide baseline diarrhea levels or complete numerical outcomes in a manner that enables effect-size synthesis; for example, in a low-dose berberine study (30 mg/kg feed for 2 weeks) conducted in unchallenged weaned piglets, clinical diarrhea outcomes were not the primary focus and baseline diarrhea reporting was not fully provided for direct comparison [79]. Ultimately, clearer baseline reporting and effect-size presentation should be strengthened alongside mechanistic and safety frameworks before plant extracts are positioned as broadly effective solutions across diverse weaning diets and management conditions.

### 4.2. Safety, Residues, Interactions and Dose-Risk Considerations (By Extract Type)

Safety remains a central translational constraint for positioning plant extracts as practical alternatives to antibiotics or pharmacological ZnO. Most piglet studies are conducted over short post-weaning windows and report limited safety endpoints, so “no adverse effects observed” typically reflects short-term tolerance under a specific diet matrix, dosage, and extract specification rather than comprehensive toxicological certainty [81,82]. Residue-related evidence is also scarce in efficacy-focused trials: validated marker-compound analytics, tissue distribution/depletion kinetics, and metabolite profiling are rarely reported, despite the plausibility of absorption and tissue distribution for lipophilic constituents (e.g., essential oils) and certain alkaloids [83,84]. Longer-term safety evidence is similarly limited, and broader panels (clinical chemistry, organ histopathology, and developmental endpoints where relevant) are needed to support repeated or sustained use [81,82]. Genotoxicity-informed assessment is particularly important for classes with hazard alerts; for example, despite genotoxicity concerns reported for sanguinarine, tolerance data for Macleaya cordata–based feed additives indicate that inclusion up to 0.75 mg sanguinarine/kg complete feed for 42 days did not adversely affect growth performance or blood biochemical indices in weaned piglets [84,85].

Interaction and dose risks are also relevant under commercial conditions where acidifiers, probiotics, enzymes, minerals, and phytogenics are frequently combined [81]. Polysaccharides can shift fermentability and SCFA profiles in a diet-dependent manner; polyphenols/tannins may bind proteins and minerals and affect palatability or nutrient utilization at higher inclusion; and essential oils or alkaloids may influence microbial susceptibility and host xenobiotic handling, creating context-dependent benefits or constraints [81,83]. Delivered exposure can deviate from formulated dose due to batch variability and losses during processing/storage, especially for volatile or thermolabile compounds, complicating the definition of safe upper limits [82]. Because safety considerations are not uniform across extract classes, Table 5 summarizes the tested inclusion ranges reported in this review, known or plausible risks, and the key evidence gaps that should be addressed before these products can be positioned as robust replacements for antibiotics or ZnO.

**Table 5:** Extract-type-specific safety considerations, tested inclusion ranges, and evidence gaps for phyto-genic extracts discussed.

Extract Type	Short-Term Tolerance/Safety Notes	Key Risks and Considerations	Key Gaps for Translation
Polysaccharides (e.g., LBP, MLP, ABPS, IRP, APS/GPS, fermented purslane)	No adverse effects were typically reported within short post-weaning windows; safety panels were usually limited (often growth/performance-focused).	Diet-dependent fermentability can shift SCFA profiles; outcomes may change with basal diet and co-additives. Potential interactions under practical diets (acidifiers, probiotics, enzymes, minerals). Batch/processing variability can alter delivered exposure.	Standardized extract specification and marker compounds; broader clinical chemistry/organ assessments in longer-term studies. Interaction testing in realistic combination programs. Clarify upper safe limits across diet matrices.
Polyphenols (flavanones/flavonoids, phenolic acids, tannins; e.g., PPP, CGA, quercetin, tannin blend, Omnivin/ALSOK)	Short-term trials rarely reported adverse effects, but safety endpoints were limited and often not extract-standardized.	At higher inclusion, polyphenols/tannins may bind proteins/minerals and affect palatability or nutrient utilization (dose-dependent risk). Variable bioavailability complicates residue prediction; residue analytics are generally NR.	Residue markers and depletion kinetics; long-term metabolic/hepatic safety. Define class-specific safe upper limits considering nutrient-binding effects. Standardize profiling (active markers, contaminants) for comparability.
Essential oils (e.g., tea tree oil, thymol/carvacrol blends; plant oils such as rice bran oil used as EO-like bioactives)	Most studies reported short-term tolerance, but comprehensive toxicology panels and withdrawal-related assessments were uncommon.	Lipophilic/volatile constituents: delivered dose can deviate due to processing/storage losses; encapsulation changes exposure. Absorption and tissue distribution are plausible; validated residue analytics are scarce. Potential interactions with other antimicrobials/additives and host xenobiotic handling are largely untested.	Pharmacokinetics and tissue residue/withdrawal studies. Longer-term organ safety and endocrine/metabolic endpoints. Quality control (chemotype/marker compounds) + stability testing under feed-manufacturing conditions.
Alkaloids (e.g., berberine; Macleaya cordata extract; PSE)	Efficacy studies are short-term; one low-dose berberine trial reported no major changes in morphology/microbiota/SCFAs, but clinical diarrhea baselines were not emphasized. Dose-dependent effects have been noted for berberine on intestinal morphology in weaned pigs.	Residue risk is plausible (systemic exposure/metabolites reported for berberine); tissue depletion generally NR. Genotoxicity-informed assessment is relevant for sanguinarine-containing products; safe use depends on specification and exposure. Potential interactions via microbial susceptibility and host xenobiotic handling.	Comprehensive residue/withdrawal and long-term hepatic/renal safety. Define safe dose ceilings with standardized extract specs (active alkaloids, impurities). Interaction studies with ZnO alternatives, antibiotics, acidifiers, and probiotics under field-relevant conditions.

Note: Dose ranges summarize short-term piglet studies included in this review (typically 14–35 d post-weaning unless otherwise stated). Safety conclusions are limited by sparse reporting of toxicology endpoints; “NR” indicates not reported in the reviewed piglet efficacy studies.

### 4.3. Product Development and Field Translation (From Compounds to On-Farm Performance)

Even when biological efficacy is demonstrated, translating plant extracts into consistent on-farm outcomes is challenged by compositional complexity, batch variability, and stability losses. The phytochemical profile of an extract can vary substantially with geographical origin, cultivar, harvest stage, and extraction conditions, which complicates reproducibility across studies and commercial production. Moreover, many bioactives—especially volatile or thermolabile constituents—may degrade during feed processing and storage, creating uncertainty in the delivered dose and, consequently, in efficacy. These constraints are widely recognized as practical bottlenecks for plant-derived antimicrobials and phytogenic additives, alongside the need to address stability and safety in application settings [41] and broader deployment challenges of phytogenic feed additives [86]. Future work should prioritize stronger quality control pipelines (e.g., compositional profiling and quantitative marker compounds), stability testing under realistic feed-manufacturing conditions, and transparent reporting of extract specifications to improve comparability across trials.

A related translational issue is bioavailability and formulation performance. Many plant-derived compounds undergo rapid degradation, biotransformation, or clearance within the gastrointestinal tract, limiting the persistence of biological effects. Polyphenols are a clear example: their structural diversity strongly influences absorption and metabolism, complicating in vivo efficacy prediction and cross-study consistency [87,88]. Formulation technologies—such as coating and encapsulation—can mitigate these limitations by improving stability and targeted release. In weaned pigs, tea tree oil (TTO) supplementation has been associated with improvements in performance and gut health indicators [66], and encapsulation approaches can further enhance outcomes, as shown by three-layer encapsulated TTO improving growth performance, antioxidant capacity, and intestinal microbiota profiles [67]. Importantly, field-scale evidence supports the translational value of microencapsulation: under commercial conditions, dietary microencapsulated organic acids plus essential oils administered during lactation significantly improved litter outcomes, including a reduction in the number of piglets with diarrhea per litter, and extending supplementation into the nursery period improved growth performance (ADFI, ADG, and FCR) [89].However, these formulation strategies also introduce cost and scalability constraints, which should be explicitly evaluated alongside biological endpoints to support realistic adoption in commercial production.

Finally, effective implementation requires clearer positioning and integration of plant extracts within broader non-antibiotic strategies for PWD control. Probiotics, organic acids, and other functional additives are already widely used, yet their efficacy can be context-dependent and influenced by diet composition and farm conditions. From an antioxidant and anti-inflammatory perspective, probiotics can contribute to diarrhea mitigation and gut resilience [33], while combination strategies (e.g., integrating acidifiers, probiotics, and essential oils) have shown promise in improving growth performance and barrier integrity in weaned piglets [70]. Given the ongoing need for antibiotic alternatives in animal husbandry [90], future research should move toward (i) well-designed comparative and combination trials with standardized endpoints (diarrhea incidence/severity, pathogen load, barrier integrity, and growth performance), (ii) robustness testing across diverse commercial settings, and (iii) translational pipelines that connect mechanistic insight to product standardization and field validation. In parallel, discovery-oriented approaches (e.g., multi-omics-guided screening of candidate actives and mechanism-informed formulation) can help identify which phytochemicals deliver reproducible benefits and under which dietary and management contexts they are most effective.

## 5. Conclusions

**Figure 1:**
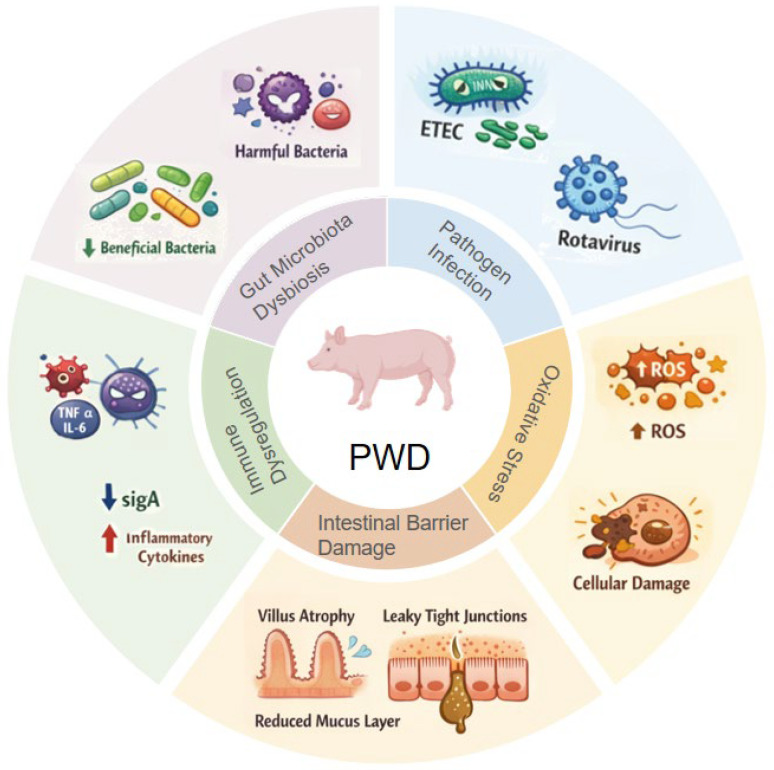
Schematic illustration of the major pathogenic mechanisms underlying PWD in piglets.

PWD remains a multifactorial challenge in pig production, arising from the combined effects of weaning stress, intestinal barrier disruption, immune dysregulation, microbiota perturbation, and enteric pathogens such as ETEC (Figure 1). In the context of tightening constraints on pharmacological zinc oxide and continued antimicrobial-resistance pressure, plant extracts represent an increasingly relevant, non-antibiotic option for supporting piglet intestinal health.

Overall, the evidence reviewed indicates that plant-derived bioactives—including polysaccharides, polyphenols, essential oils, and alkaloids—can reduce PWD risk and severity through complementary mechanisms. Reported benefits commonly include improved antioxidant capacity, moderated inflammatory responses, enhanced epithelial integrity, suppression of pathogen growth or virulence, and rebalancing of gut microbial communities, ultimately strengthening endogenous defenses during the weaning transition.

To enable consistent real-world implementation, future research should move beyond associative efficacy reports toward clearer causal and mechanistic validation, supported by harmonized clinical and functional endpoints. In parallel, rigorous safety evaluation and dose–response characterization are needed to define effective and safe inclusion ranges across realistic feeding durations and production contexts. Finally, translation to commercial practice will require product-oriented solutions that improve standardization and batch consistency, preserve bioactivity through feed processing and storage, and optimize practical delivery to achieve reliable on-farm performance.

## Data Availability

The original contributions presented in this study are included in the article. Further inquiries can be directed to the corresponding author.
